# Mortality Rates of Coronavirus Disease 2019 (COVID-19) Caused by the Novel Severe Acute Respiratory Syndrome Coronavirus-2 (SARS-CoV-2)

**DOI:** 10.7759/cureus.14081

**Published:** 2021-03-24

**Authors:** Venkataramana Kandi, Sudhakar Thungaturthi, Sabitha Vadakedath, Rajkumar Gundu, Ranjan K Mohapatra

**Affiliations:** 1 Clinical Microbiology, Prathima Institute of Medical Sciences, Karimnagar, IND; 2 Biochemistry, Chalmeda Anandrao Institute of Medical Sciences, Karimnagar, IND; 3 Biochemistry, Prathima Institute of Medical Sciences, Karimnagar, IND; 4 Chemistry, Government College of Engineering, Keonjhar, Keonjhar, IND

**Keywords:** covid-19, sars-cov-2, pandemic, statistics, infection fatality rate (ifr), case fatality rate (cfr), world bank countries, who regions, age, comorbidities

## Abstract

Background

The significance of the global prevalence and incidence of coronavirusdisease 2019 (COVID-19) is a measure of its severity. However, without statistical data, one cannot understand the novel severe acute respiratory syndrome coronavirus-2 (SARS-CoV-2) pandemic. This study, based on good data, enables us to know how the disease is spreading, what impact the pandemic has on the lives of people around the world, and whether the countermeasures that countries have been taking are successful for controlling and preventing the disease. Therefore, this study is undertaken to estimate the infection fatality rates (IFRs) and case-fatality rates (CFRs) in various countries and regions of the world.

Methods

COVID-19-related data were collected from various countries belonging to different World Bank categories based on economies (low-income, low-middle income, upper-middle income, and high-income countries) and the World Health Organization's (WHO's) regional classification of countries (the Americas, European, African, South-East Asia, Eastern Mediterranean, and Western Pacific regions). The data were collected from the WHO’s dedicated website on COVID-19, and statistical methods like mean, standard deviation, p-value, and percentages were used to calculate the IFR and CFR.

Results

Mexico (8.94%) reported the highest IFR among all the countries. The low-income countries reported increased IFR (2.46±1.91) as compared to the other groups. The European region (7.3%) and the American region (5.3%) recorded the highest CFRs. The South-East Asian region reported the lowest CFR (1.1%).

Conclusions

The low-income group countries showed higher rates of IFR and lower CFRs. Lower IFRs and increased CFRs were noted among the high-income group countries and the American and European regions respectively. The varied IFRs and CFRs could be attributed to multiple factors that include climatic conditions, living environments, age, sex, comorbidities, among others.

## Introduction

Severe acute respiratory syndrome Coronavirus-2 (SARS-CoV-2) is a novel CoV that has spread throughout the world and is responsible for coronavirus disease 2019 (COVID-19) [1]. There is currently no specific therapeutic agent to treat COVID-19 [2-3]. Preventive vaccines with variable efficacies have recently been made available in most parts of the world. Due to the increased infection rates and the movement of people within and across the countries, several nations are facing the second and third waves of COVID-19 [4]. Currently, the morbidity and mortality among the world population attributed to the pandemic is a cause for serious concern [5].

Extensive research is presently undertaken across the world in the search for an effective anti-viral agent/vaccine to cure and prevent the dreaded disease. The production of vaccines and vaccinating people, which was previously perceived as a mirage in the desert, has currently been realized. The disease in humans is at its peak in terms of the spread deep into the community. COVID-19 can be effectively contained by following precautionary measures like improved hygiene and sanitation, along with safe lifestyle practices like wearing face masks, face shields, and other personal protective equipment (PPE), which can help decrease the spread of the viral infection. Having seen the pandemic for over a year now, there remains a lot to be explored about the other potential modes of virus spread. An improvement in living conditions, such as access to clean water, improved sewerage systems, better food, and personal hygiene conditions (such as hand washing), can significantly minimize the spread of the virus and the incidence of infection [2].

COVID-19 transmission, morbidity, and mortality are noted to be variable among the affected countries. The World Health Organization (WHO) has been constantly collecting data and maintaining a dedicated website that presents statistics related to the rates of infection, recoveries, and mortality in the present situation [6]. The pandemic looks like an ongoing and far from ending process that may continue until the cases cease to appear among the people around the world. There are continuous reports of death among infected people, and several countries have been noticing flares of infection transmissions, forcing the respective countries/regional governments to restrict the movement of people by enforcing various levels of lockdowns and other measures to control the spread of infection. Also, we have seen reports of differences in the mortality rates of COVID-19 across the world [7]. The present study is aimed to assess the mortality rates (infection fatality rate (IFR), and case fatality rate (CFR)) attributed to COVID-19 among the World Bank classification of countries based on economies (low-income, low-middle income, upper-middle income, and high-income countries) and the WHO’s region-wise classification (the Americas, European, African, South-East Asia, Eastern Mediterranean, and Western Pacific regions) [8-9].

## Materials and methods

This statistical observational study was undertaken to assess the mortality rates of COVID-19 and compare the infection death rates among various countries as classified by the World Bank based on economies [8]. Also, the study has attempted to evaluate the IFRs and case fatality rates CFRs among the WHO’s regions [9].

Study population

The WHO’s global reports of those suffering from COVID-19 among the 190 plus affected countries all around the world were taken as the study population [6,10]. Those reports of total confirmed cases and total deaths that occurred until March 1, 2021, were used for statistical calculations.

Inclusion and exclusion criteria

All individuals diagnosed with COVID-19 infection and belonging to all age groups and different sexes were included in the study.

Infection fatality rate

The IFR was calculated using the following formula:

IFR (%)=Total deaths/Actual infectionsX100

Case fatality rate

CFR (%)=Total deaths/Clinical cases (deaths and recovered)X100

Statistical methods

Data collected from the WHO website were entered into a Microsoft Excel (Microsoft Corporation, Redmond, WA) sheet. Mean, standard deviation, p-value, and percentages were calculated.

## Results

Among the low-income countries, the Syrian Arab Republic (6.64%) and Sudan (6.12%) have reported the highest IFRs followed by Eswatini (3.83%), Malawi (3.29%), and the Democratic Republic of the Congo (3.17%). Egypt (5.89%) reported the highest IFR among the low-middle income countries, which is closely followed by Bolivia (5.30%), Tunisia (3.44%), and El Salvador (3.11%). Among the upper-middle-income group countries, Mexico (8.94%) reported the highest IFR, which probably is the highest for any country or region of the world, followed by China (4.74), Iran (3.60%), Peru (3.49%), and South Africa (3.33%). Bulgaria (4.07%) reported the highest IFR among the high-income group countries followed by Hungary (3.40%), Greece (3.28%), Italy (3.26%), Australia (3.13%), United Kingdom (2.95%), and Germany (2.85%). The cumulative IFR of the low, low-middle, upper-middle, and high-income countries is depicted in Table 1.

**Table 1:** Infection fatality rates among various economies of the world * Comparison of p-values within the group **Comparison of p-values between the groups Statistically significant if the p-value is <0.05

World Bank's income group country classification	Mean±SD	p-value	High-income	Upper-middle income	Low-middle income	Low-income
Low-income	2.46±1.91	0.198*	0.070**	0.532**	0.240^**^	-
Low-middle income	1.91±0.99	0.054*	0.558**	0.584**	-	0.240**
Upper-middle income	2.11±1.65	0.201*	0.222**	-	0.584**	0.532**
High income	1.77±1.01	0.079*	-	0.222**	0.558**	0.070**

In the WHO’s region-wise statistical observation of six regions, the IFR was the same (0.02%) for the American, European, and eastern Mediterranean regions. The South-East Asian and the Western Pacific regions had similar IFR (0.01%). The European region showed the highest CFR (7.3%) followed by the American region (5.3%). The South-East Asian region recorded the lowest (1.1%) CFR. The details of IFRs and CFRs concerning the WHO’s regions are presented in Table 2.

**Table 2:** The WHO’s region-wise IFR and CFR percentages WHO: World Health Organization; IFR: infection fatality rate; CFR: case fatality rate

WHO’s region classification	IFR (%)	CFR (%)
American region	0.02	5.3
European region	0.02	7.3
African region	0.02	1.9
South-East Asia region	0.01	1.1
Eastern Mediterranean region	0.02	2.2
Western Pacific region	0.01	1.8

## Discussion

Coronavirus

**Figure 1:**
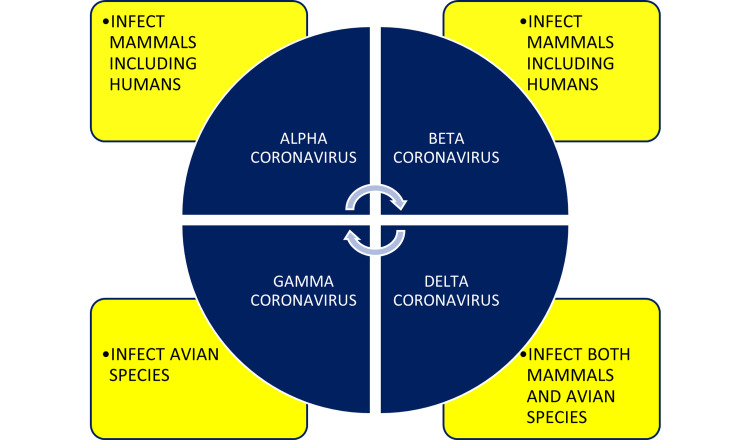
The coronavirus genera and host preferences

The word “corona” in coronavirus means a crown, wreath, or garland. The corona of the sun's surface has the same meaning as a halo in nature. The corona of the sun is considered a more heated surface than the inside of the sun. Similarly, the novel CoV is spreading COVID-19 rapidly and putting the healthcare systems throughout the world under increased stress. An epidemic happens when a disease spreads between large numbers of people within a country in a short period. When an epidemic goes global or crosses countries, it is called a pandemic. The novel CoV belongs to the family *Coronaviridae*. It is an enveloped, positive-sense, single-stranded ribonucleic acid (RNA) virus. The coronaviruses are classified into four genera as shown in Figure 1 [11].

**Figure 2:**
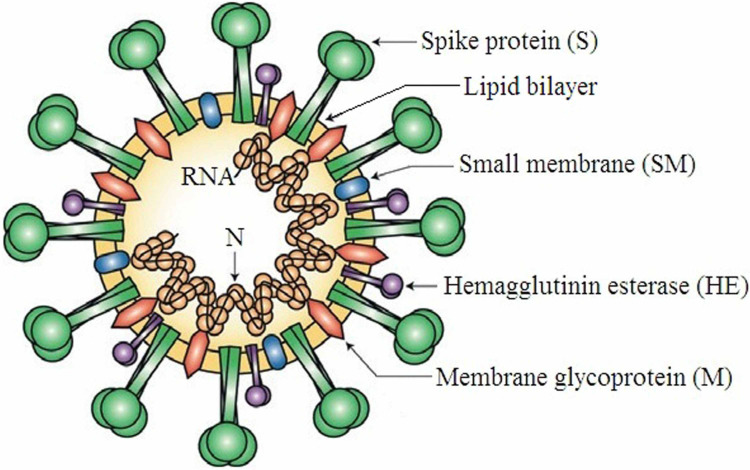
The diagrammatic representation of the structure of coronavirus Reproduced with permission from Reference [1]. Main source: Finlay BB, Hancock RE. Can innate immunity be enhanced to treat microbial infections? Nature Reviews Microbiology, 2004, 2: 497–504. © Springer Nature

They have a large genome (27 to 32 Kb) packed inside a nucleocapsid (capsid protein N) and surrounded by an envelope. The envelope has three structural proteins: the membrane glycoprotein (M), the envelope protein (E), and the spike protein (S). The M, E, and S proteins help the virus in assembly, mediate the entry of the virus into host cells, and are a major inducer of host immune responses, respectively, as depicted in Figure 2 [1,11-12].

The novel CoV is presumed to be a zoonotic virus that accidentally crossed over to humans [5]. Infection to the host cell is initiated by the attachment of spike proteins to the host cell receptors. After attachment, the host cell protease enzyme cleaves the spike proteins, which, in turn, allows the entry of the virus by endocytosis or sometimes by direct fusion of the viral envelope with the host membrane. The viral proteins are translated directly from its genome by the host cell ribosomes since the virus has a 5’ cap (methylated) and a 3’ polyadenylated tail that acts as messenger RNA. Thus, the infected hosts shed the virus through respiratory secretions and aerosols into the environment, and transmission occurs from one host to another. The novel CoV infects the epithelial cells of the respiratory tract and the mucosa of the nose and eyes [1,11-12].

WHO's region-wise classification revealed an overall IFR of 0.1% across the world population. This was significantly lower than a previous report (0.68%), which was done using the data collected from six months into the pandemic [13]. The results of the present study showed the highest IFR among the low-income group countries (2.46±1.91) and the lowest among the high-income countries group (1.77±1.01).

The cumulative CFR for all the WHO regions was found to be 3.26%. The CFRs revealed the European region (7.3%) to be the most affected followed by the American region (5.3%) [14-15]. The increased CFR may be, in part, explained by the fact that the burden of the aged population is high among various countries belonging to these regions of the world. Also, the high CFR in the European regions may be attributed to age and the climatic conditions (subtropical and temperate) in these regions that favor the survival of the virus and increased transmissibility.

The South-East Asian Region (SEAR) showed the least CFR (1.1%). Although no definitive cause may be attributed to a considerably low CFR in SEAR, the hot and humid environmental conditions could have affected the virus's survival. An efficient immune response among the people in clearing the infection, probably facilitated by increased exposure to microbes because of overcrowding and BCG (bacillus Calmette-Guerin) vaccination may have contributed to lower CFRs [16]. A few recent studies have found an association of CFR with increased age, comorbidities, demographic characteristics, and socio-economic factors [17-18].

The age groups and the cumulative death rates among people infected with the novel coronavirus showed >80 years, 14.8%; <80 to 50 years, 12.9%; and <50 years, 1.0%, respectively. Also, it was noted that individuals with an age greater than 65 years accounted for 56%-62% of deaths among the COVID-19 patients [19-24].

The elderly age is at increased risk for COVID-19-related complications due to immune senescence and comorbidities. This promotes a virus-induced 'cytokine storm' resulting in life-threatening respiratory failure and multisystem involvement. The aggressive inflammatory response of the host to SARS-CoV-2 virus infection releasing large amounts of proinflammatory cytokines is known as a cytokine storm [25].

The older aged people with varied comorbidities succumb to death proportionately due to signs of sepsis and intravascular coagulation. This was attributed to the nucleocapsid protein of the virus that plays an important role in the pathogenesis of COVID-19-related coagulation disorders [26].

In the general population, more than 80% of those who get infected will recover from the disease without needing hospital admission and treatment. Around one out of every five persons who get COVID-19 becomes seriously ill and develops difficulty in breathing. The common cause for the spread of the virus could be in the form of community transmission, cluster cases, and sporadic patterns of spread. The data from clinical and virologic studies that have been collected by repeated biological samples from confirmed patients provide evidence that shedding of SARS-CoV-2 is highest in the upper respiratory tract (nose and throat) early in the course of the disease. That is, within the first three days from onset of symptoms, which at present takes around four to seven days on an average for infection [1,11-12]. The incubation period is the time gap between exposure and the development of clinical symptoms, wherein the infected persons are more contagious. It was also observed that shorter incubation periods of the virus indicate high infective capacity, greater replication in the host, as well as more aggressive and damaging inflammatory response leading to the severe form of the disease.

The WHO recommends global surveillance of SARS-CoV-2 infections to contain the virus at the earliest by following certain objectives that include monitoring the trends of COVID-19 at national and international levels. Other measures suggested by the WHO include rapidly detecting new cases in countries where the virus is not circulating, monitoring of cases and contacts in countries where the virus has started to circulate within the community, conducting risk assessments at the national, regional, and global levels, and providing epidemiological information to guide preparedness and response measures [27].

The main concern during the current pandemic is to minimize the morbidity and mortality related to COVID-19 by determining the treatment plan, predicting the disease course and expected outcomes, evaluating and predicting the potential for virus spread, identifying susceptible individuals, vaccinating and tracing the movement of the virus through the community, and worldwide surveillance.

The statistical observations have been instrumental in evaluating the potential of the novel CoV to cause COVID-19-related severe complications among aged people with comorbidities and men. The restricted movement of the people and closure of educational institutions in developing countries like India helped in controlling the spread of infection. Awareness programs by the government on the usage of face masks, social distancing, frequent hand sanitization, and the advantages of taking immunity-enhancing food like nuts, sprouts, fruits, and vegetables may help in minimizing the morbidity and mortality caused by COVID-19.

The aggregate CFR observed in this study (3.26%) was found to be higher than the previous 1918s' Spanish flu pandemic (>2.5%). The previous influenza virus* *pandemics showed a CFR of <0.1% [28-29]. Also, it is interesting to note that the previous SARS-CoV and Middle-East Respiratory Syndrome (MERS) CoV pandemics had CFRs of >10% and up to 35%, respectively. Both these pandemics were restricted to few countries and had become invisible thereafter [30]. Therefore, the current SARS-CoV-2 pandemic is probably the worst pandemic ever, which has affected most parts of the world and has already lasted over a year, with high CFRs.

The present statistical observational study of IFRs and CFRs in COVID-19 is to show the significance of the disease in countries around the world in terms of the severity of the disease among the population. The mortality rates varied in different parts of the world. This could be attributed to the type of spread of the disease: scattered, sporadic, or community spread. Also, variations in disease outcomes are attributed to the climatic conditions, age, gender, and presence of comorbidities and coinfections in the patients.

Limitations

Although the data and statistics presented in this study are based on the WHO website, we must accept the fact that even the best available data on the COVID-19 pandemic may still be far from perfect.

## Conclusions

Statistical data play a major role in understanding and controlling the COVID-19 pandemic. Although vaccines have been rolled out, complete immunization of people across the globe may take a significant amount of time. Data collection and analysis remain essential tools to understand disease transmission and clinical outcomes. It is necessary to make statistical inferences to assess the percentage of people who become infected during a disease outbreak and how many of them eventually recover or succumb to the disease. To effectively control the pandemic, regular reporting, preferably daily data on how many confirmed cases and total deaths due to the novel CoV disease, from every affected region of the world assumes increased significance. Regular monitoring of mortality rates, increased testing, observing the trends of infection within a country and across the globe, and regularly revising infection control practices in tune with the available up-to-date pandemic-related information is the need of the hour. Marie Curie once said, “Nothing in life is to be feared, it is only to be understood. Now is the time to understand more, so that we may fear less.” This quote appears well-suited to the current COVID-19 pandemic.
